# Does “Overall Catastrophic Health Care Expenditure” Make Sense?

**Published:** 2018-08

**Authors:** Kamran BAGHERI LANKARANI, Sulmaz GHAHRAMANI

**Affiliations:** Health Policy Research Center, Institute of Health, Shiraz University of Medical Sciences, Shiraz, Iran

## Dear Editor-in-Chief

We read with interest the article on overall catastrophic health expenditure (CHE) in Iran by Aryankhesal et al (1). CHE is a thought-provoking subject interrelated to out of pocket (OOP) payment. A prosperous health financing system should protect households from high OOP payments when they are ill. OOP payment as the most inequitable way for financing in health system, expose households to CHE. CHE may disrupt living standards of households. This situation could be prevented with risk protection financial policies, even in poor households and one of the major goals in universal health coverage proposed by WHO is to reduce CHE and OOP (2, 3).

CHE has raised national health policy concerns in Iran in recent years. In the fourth national development plan of Iran launched in 2005, the aim of reduction of CHE to < 1% was proposed (4). As there is wide range of values for CHE in different studies, Aryankhesal et al., in their systematic review tried to estimate one summarized convincing and reliable evidence measure that represents the overall percentage of households faced with CHE in Iran between 1984 to 2014 (5).

There are several concerns on this systematic review. Heterogeneity is one of the most disputed aspects of many systematic reviews and could explain how confidently authors could draw overall conclusion. The interpretative problems depend on magnitude of heterogeneity that determines the extent to which it might influence the conclusions of the meta-analysis. Authors have used two different ways for evaluation of heterogeneity, Q-test which informs about the presence of heterogeneity and *I*^*2 *^values for the extent of such heterogeneity (6–8). Using random effect model, almost all estimated household’s CHE in this review including overall and subgroups estimations (community center, hospital, rural and urban) had high and significant Q and I^2^ were more than 85 % except for hospital costs (with a lower I^2^).

This high heterogeneity precludes meta-analysis. A broader view of social and geographic factors which might have caused this variability, as well as different definitions used in for CHE, were required. Further, the authors did not provide I^2^ with correspondent confidence intervals (8) which will better define the heterogeneity. There was a need for detailed describing of distribution of studies (urban, rural, hospital), methodology and definition of CHE used by the authors of original studies and the results for qualitative assessment since the results of excluded articles could affect estimations.

Looking to the feature the total number of studies in forest plot is not consistent with total number of included studies.

High level of heterogeneity was quite expected in estimation of CHE in Iran, but the results of this meta-analysis should be interpreted with caution and evaluation of sources of this heterogeneity is strongly recommended. Review on households’ CHE in recent years (especially after Health Transformation Plan in Iran, since 2014 till now) seems to be more interested.
